# Optimized Reinforcement Learning-Driven Model for Remote Sensing Change Detection

**DOI:** 10.3390/jimaging12030139

**Published:** 2026-03-19

**Authors:** Yan Zhao, Zhiyun Xiao, Tengfei Bao, Yulong Zhou

**Affiliations:** 1 School of Electric Power, Inner Mongolia University of Technology, Hohhot 010080, China; 20231800576@imut.edu.cn (Y.Z.); 20221100441@imut.edu.cn (Y.Z.); 2 Intelligent Energy Technology and Equipment Engineering Research Center of Colleges and Universities of Inner Mongolia Autonomous Region, Inner Mongolia University of Technology, Hohhot 010080, China; 3 Inner Mongolia Key Laboratory of Electrical and Mechanical Control, Inner Mongolia University of Technology, Hohhot 010080, China; 4 School of Electronic Information and Electrical Engineering, Shanghai Jiao Tong University, Shanghai 200240, China

**Keywords:** remote sensing change detection, deep learning, reinforcement learning, U-Net, adaptive segmentation, UAV dataset

## Abstract

In recent years, deep learning has driven remarkable progress in remote sensing change detection (CD); however, practical deployment is still hindered by two limitations. First, CD results are easily degraded by imaging-induced uncertainties—mixed pixels and blurred boundaries, radiometric inconsistencies (e.g., shadows and seasonal illumination changes), and slight residual misregistration—leading to pseudo-changes and fragmented boundaries. Second, prevailing methods follow a static one-pass inference paradigm and lack an explicit feedback mechanism for adaptive error correction, which weakens generalization in complex or unseen scenes. To address these issues, we propose a feedback-driven CD framework that integrates a dual-branch U-Net with deep reinforcement learning (RL) for pixel-level probabilistic iterative refinement of an initial change probability map. The backbone produces a preliminary posterior estimate of change likelihood from multi-scale bi-temporal features, while a PPO-based RL agent formulates refinement as a Markov decision process. The agent leverages a state representation that fuses multi-scale features, prediction confidence/uncertainty, and spatial consistency cues (e.g., neighborhood coherence and edge responses) to apply multi-step corrective actions. From an imaging and interpretation perspective, the RL module can be viewed as a learnable, self-adaptive imaging optimization mechanism: for high-risk regions affected by blurred boundaries, radiometric inconsistencies, and local misalignment, the agent performs feedback-driven multi-step corrections to improve boundary fidelity and spatial coherence while suppressing pseudo-changes caused by shadows and illumination variations. Experiments on four datasets (CDD, SYSU-CD, PVCD, and BRIGHT) verify consistent improvements. Using SiamU-Net as an example, the proposed RL refinement increases mIoU by 3.07, 2.54, 6.13, and 3.1 points on CDD, SYSU-CD, PVCD, and BRIGHT, respectively, with similarly consistent gains observed when the same RL module is integrated into other representative CD backbones.

## 1. Introduction

As a fundamental task in remote sensing image analysis, change detection aims to identify surface changes from multi-temporal imagery acquired over the same geographic area and serves as a key enabling technique for applications such as dynamic land-use monitoring, disaster assessment and response, urban expansion analysis, and ecological environment evaluation [1,2]. With the continuous increase in observation frequency and spatial resolution, practical deployments demand change detection methods that are not only accurate and efficient but also capable of maintaining stable generalization under complex scenes, varying imaging conditions, and cross-region transfer. Conventional methods rely on manual interpretation or handcrafted features (e.g., differencing, ratioing, and PCA) and often exhibit limited robustness under complex terrains, illumination variations, and highly spectrally similar backgrounds, making them inadequate for large-scale automated processing.

In recent years, deep learning has been widely adopted for remote sensing change detection. By building end-to-end neural networks (e.g., Siamese architectures, dual-branch U-Net variants, and multi-scale feature fusion models), these approaches can learn discriminative representations from bi-temporal imagery and substantially improve the level of automation and overall accuracy [3,4,5,6]. However, in complex remote sensing scenarios, existing deep learning–based change detection methods still face several major challenges. First, change detection typically operates under severe class imbalance and cluttered backgrounds; predictions are easily affected by local textures, shadows, seasonal variations, and slight residual misregistration, resulting in pseudo-changes. Second, when change boundaries are ambiguous, object scales vary, or noise/interference is strong, a single-shot forward inference often fails to achieve both reliable boundary delineation and detail preservation. Third, although networks can fit a predefined loss during training, their inference behavior remains largely “static”: once a prediction is produced, the model lacks an explicit feedback mechanism to adaptively correct errors, and the errors tend to accumulate under unseen scenes, imaging-condition shifts, or novel object patterns. In other words, the bottleneck is not merely representation capacity but the absence of feedback-driven self-correction during inference—an ability that is particularly critical for boundary- and pseudo-change–sensitive change detection.

To address these issues, prior work has explored multiple directions. Boundary-aware losses (e.g., Boundary Loss, Hausdorff Distance Loss, and edge-aware Dice loss) explicitly constrain boundary errors to enhance edge delineation [7,8,9]; graph-based post-processing such as conditional random fields (CRFs) improves spatial coherence [10]; and architectural innovations based on vision Transformers, state-space modeling (e.g., Mamba), or large foundation models strengthen global dependency modeling and multi-scale representations for complex scenes [11,12,13]. Recently, Transformer-based change detection models (e.g., BIT, ChangeFormer, and SwinSUNet) [14,15,16] and state-space–model–based variants (e.g., Mamba-CD, AM-CD and CDMamba) [17,18,19] have become active research topics and achieved promising performance. Nevertheless, most of these improvements still follow a static optimization paradigm: models are trained to optimize a fixed objective, and inference remains a one-pass prediction process. In difficult local regions caused by strong noise, weak boundaries, or residual misregistration, an explicit “prediction–feedback–correction” iterative procedure is typically absent. Therefore, enabling change detection models with dynamic, self-adaptive optimization capability beyond static inference remains an important open problem.

The above limitations motivate researchers to explore deep reinforcement learning (RL) for visual segmentation. RL is characterized by a closed-loop mechanism of “state–action–reward–update”, which naturally models feedback-driven sequential decision-making and iterative optimization and thus provides a principled route to correct local errors that cannot be adequately handled by a single forward pass. Existing RL-based studies in visual segmentation suggest that sequential decision-making and iterative refinement are well suited for boundary refinement and local error correction. For example, Duan et al. proposed RL-CoSeg by formulating co-segmentation as a Markov decision process (MDP) and iteratively improving foreground boundary quality via RL [19]. Furuta et al. introduced pixelRL, a pixel-wise multi-step RL framework for image processing tasks such as denoising and enhancement, where an action sequence progressively adjusts pixel-level representations [20]. Han et al. modeled video object segmentation as an MDP and learned a cutting-agent to perform multi-step segmentation decisions, adapting to evolving targets in temporal sequences [21]. Vecchio et al. further explored multi-agent formulations for video object segmentation and interactive feedback modeling, enabling policy updates to better absorb feedback from different sub-tasks and local regions [22]. Collectively, these works demonstrate that RL can explicitly incorporate “error feedback” into segmentation optimization, providing methodological evidence for pixel-level iterative correction in remote sensing change detection [22,23,24,25,26,27].

Remote sensing change detection differs from the above segmentation settings: it relies on bi-temporal discrepancies and is affected by misregistration, seasonal/illumination changes, spectral ambiguity, and strong class imbalance [28,29,30,31]. Accordingly, the RL state, actions, and rewards must be designed specifically for these factors [32]. Although RL-based change detection is still nascent, its feedback-driven refinement naturally supports boundary sharpening, pseudo-change suppression, and scene-adaptive generalization beyond static inference [33,34,35].

Motivated by these considerations, this paper proposes a hybrid change detection framework that integrates a dual-branch U-Net with deep reinforcement learning to achieve feedback-driven iterative refinement of the initial change probability map [36]. The dual-branch U-Net extracts multi-scale representations and produces an initial change probability map, while the RL module organizes multi-scale fused features, confidence/uncertainty cues, and spatial consistency information into the state and outputs pixel-level corrective actions through a learned policy. By refining the probability map over multiple steps, the proposed framework enhances boundary delineation and suppresses pseudo-changes [37]. Different from fixed-rule post-processing (e.g., CRF or morphology), the RL module is formulated as an MDP and learns a generalizable correction policy via reward feedback, enabling the model to adaptively select correction directions and magnitudes according to region structures and prediction uncertainty.

From an imaging and interpretation perspective, the introduced RL module is not merely used to “improve classification accuracy” but can be viewed as a feedback-driven, self-adaptive optimization mechanism for interpretation quality. After the deep network produces an initial change probability map, we regard it as a posterior estimate of the underlying change distribution, and the RL agent performs pixel-wise corrections through multi-step iterations [38]. The optimization objective corresponds to more intrinsic interpretation-quality dimensions in remote sensing, including boundary fidelity, spatial coherence, and the reliability of spatio-spectral representations. The RL state explicitly incorporates confidence/uncertainty and spatial consistency constraints (e.g., neighborhood consistency, connectivity, and edge responses), allowing the agent to distinguish “reliable” from “high-risk” regions and apply differentiated correction strategies. In this way, the framework adaptively suppresses imaging uncertainties such as blurred boundaries, shadow interference, texture similarity, and slight residual misregistration. Moreover, the feedback-driven iterative refinement is extensible: when sensor resolution, noise levels, or spectral channels vary, richer spatio-spectral consistency cues and sensor-related constraints can be integrated into the RL state and reward. In this work, we first validate the effectiveness of the proposed framework under an RGB input setting, providing a foundation for future extensions to multi-spectral or multi-resolution scenarios.

In summary, the main contributions of this work are threefold:

(1) We introduce a PPO-driven pixel-level probabilistic iterative correction mechanism for remote sensing change detection, enabling dynamic refinement of static initial predictions.
(2) We design a change-detection–oriented RL formulation with state representations that integrate multi-scale features, confidence/uncertainty cues, and spatial consistency signals, allowing adaptive refinement in boundary-ambiguous and pseudo-change–prone regions.
(3) We validate the proposed framework on multiple public benchmarks and a real-world PV change detection dataset, and provide comparative and ablation analyses to quantify the contributions of key components.

## 2. Materials

### 2.1. Datasets and Study Area

This study evaluates the proposed method on three public benchmarks and a newly constructed photovoltaic change detection dataset (PVCD). PVCD was collected over eight typical PV farm sites located in the Kubuqi Desert (Ordos, Inner Mongolia), a dune-dominated region on the northern margin of the Ordos Plateau. In recent years, large-scale PV stations have been rapidly deployed in this area, where PV arrays exhibit dense, regular patterns that provide representative targets for remote sensing change detection.

Change Detection Dataset (CDD): CDD is a widely used benchmark consisting of bi-temporal remote sensing image pairs with pixel-level binary labels (change/unchanged) across diverse environments (e.g., urban, cropland, water, and woodland). In this study, we used 15,598 pairs in total, with 10,000 for training and the remaining pairs split into validation and testing.

SYSU-CD Dataset: SYSU-CD contains 20,000 bi-temporal image pairs with change annotations covering diverse change patterns (e.g., building construction/demolition, road expansion, and farmland conversion). We followed the standard split of 12,000/4000/4000 for training/validation/testing.

BRIGHT Dataset: BRIGHT is an open-access [23], globally distributed, event-diverse multimodal dataset curated for AI-based disaster response. It covers five natural disasters and two man-made disasters across 14 regions worldwide, providing about 4200 paired optical and SAR images with a spatial resolution of 0.3–1 m, and includes over 380,000 building instances for fine-grained damage-related analysis. Following the official partition, we used 2890 image pairs for training and 349 pairs for testing; the remaining samples were reserved for validation in our implementation. For consistency with the RGB-only setting used throughout this study, we used the optical imagery from BRIGHT as model input.

Photovoltaic Change Detection Dataset (PVCD): UAV-based multi-temporal imagery was collected over eight PV farm areas in the Kubuqi Desert. After geometric correction and mosaicking, orthorectified images were generated (5500 × 3262 to 11,000 × 6700 pixels), cropped into 256 × 256 patches, and augmented (rotation, flipping, and brightness adjustment), yielding 11,000 high-resolution image pairs with pixel-level change labels.

To prevent data leakage caused by spatial autocorrelation, we adopted geographically disjoint splits. For CDD, SYSU-CD, and BRIGHT, we used the official train/val/test partitions. For PVCD, the validation and testing samples were collected from independent sample areas that were never used during training.

### 2.2. Data Acquisition and Preprocessing

#### 2.2.1. UAV-Based Multispectral Data Acquisition and Preprocessing

The key technical specifications of the UAV and sensors are summarized in Table 1.

The experimental data used in this study were acquired using the DJI Matrice 300 RTK industrial-grade UAV (DJI Innovations Co., Ltd., Shenzhen, China). The UAV was equipped with an MS600Pro multispectral gimbal camera (Changguang Yuchen Information Technology and Equipment Co., Ltd., Qingdao, China), which provides a spatial resolution of 1.3 megapixels and captures spectral data within the 400–1000 nm range. The MS600Pro includes six spectral bands centered at 450 nm, 555 nm, 660 nm, 720 nm, 750 nm, and 840 nm, corresponding to the blue, green, red, and three near-infrared bands, respectively. The UAV system integrates a high-precision satellite navigation module, enabling autonomous flight path planning within the study area.

**Figure 1:**
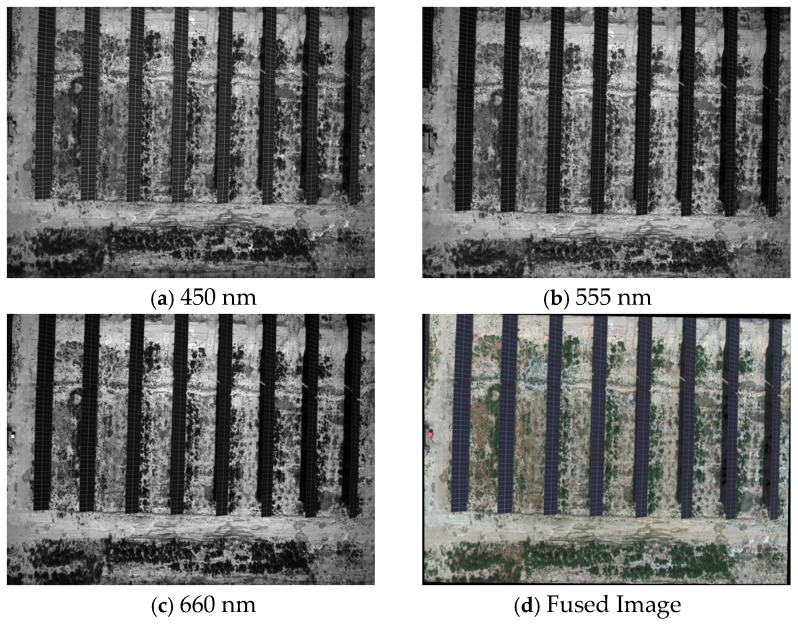
Remotely sensed image of an individual acquisition site. (**a**) Image at 450 nm, (**b**) Image at 555 nm, (**c**) Image at 660 nm, (**d**) Fused image generated from the three bands.

Data were purposefully acquired in June 2025, as this period coincides with the prevalence of stable illumination conditions, which are ideal for the operation of PV panels. Although the equipment captured both visible and near-infrared imagery, only the RGB visible bands were used for analysis in this study [39]. The core contribution of this work lies in validating the dynamic optimization capability of deep reinforcement learning (RL) for change detection—particularly in boundary refinement and pseudo-change suppression. This enhancement mechanism primarily relies on spatial geometric features and contextual semantic information, which can be adequately captured by the high-resolution texture information provided by RGB bands (e.g., contours of photovoltaic panels and building edges). Meanwhile, the publicly available datasets employed in our experiments are widely adopted RGB-only benchmarks in the change detection community; maintaining consistent RGB inputs across all datasets ensures a fair and direct performance comparison with existing state-of-the-art methods. Given that the application of RL to change detection remains an emerging research direction, this study focuses on establishing its effectiveness on RGB bands as a foundational step, with the extension to multi-spectral information reserved for future exploration [40]. Thus, all datasets uniformly use RGB bands (Figure 1). A small pilot on PVCD with RGB + NIR (reported in Figure S1 in the Supplementary Materials) indicates that the RL refinement exhibits consistent trends in boundary improvement and false-alarm reduction under both RGB-only and RGB + NIR settings.

Prior to image collection, a calibration panel provided with the camera was used to capture reference images, compensating for illumination variations caused by different times or weather conditions and reducing radiometric inconsistencies. After assembling the UAV system, the boundary of the study area was imported into the flight control software, and subregion flight routes planned based on remote sensing imagery. The flight mission parameters were set as follows: flight altitude of 120 m, forward overlap of 70%, and side overlap of 80%. Data acquisition was carried out under near-clear-sky weather conditions (cloud cover < 10%) between 10:00 AM and 14:00 PM local time to ensure image consistency by minimizing angular variations in solar illumination. The UAV flight speed was set to 11 m/s, with a ground sampling distance (GSD) of 6 cm and an image spatial resolution of 2.5 cm. An S-shaped flight path was adopted to ensure complete coverage and spatial continuity across the study area. To mitigate overlap variation caused by acceleration, deceleration during turns and wind gusts as the UAV was flying, an equidistant image capture mode was employed to enhance data stability.

**Figure 2:**
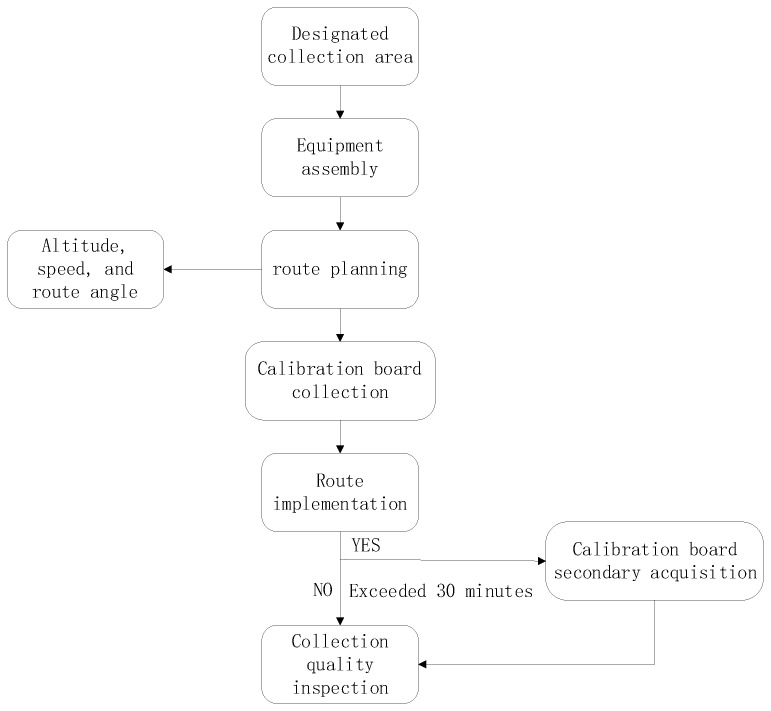
Sequence of steps that were followed to compile UAV remote sensing data.

Figure 2 shows the sequence of steps that were followed to compile UAV remote sensing data that were used in this study.

**Figure 3:**
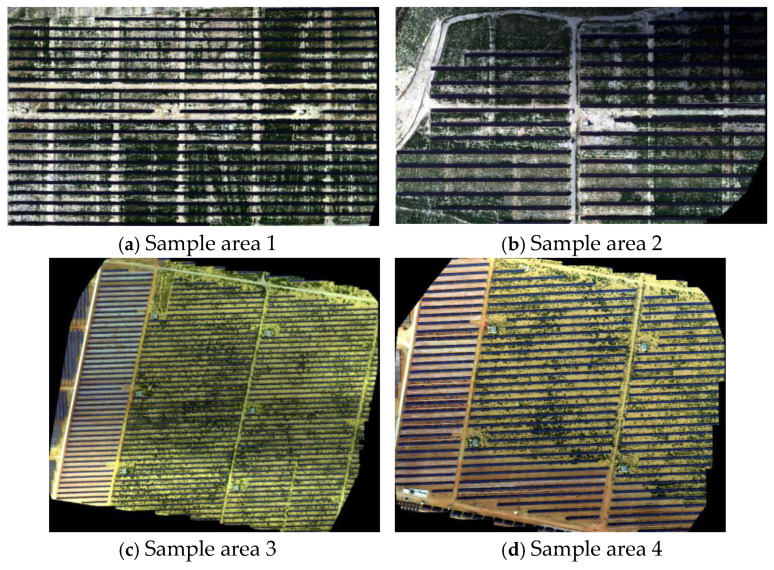
UAV-derived orthophoto coverages of the study area’s sample sites. (**a**) Sample area 1, (**b**) Sample area 2, (**c**) Sample area 3, (**d**) Sample area 4.

After data collection, the multispectral imagery was processed using the YuSense Map software (Changguang Yuchen Information Technology and Equipment Co., Ltd., Qingdao, China), which is compatible with the MS600Pro gimbal camera. This software was used for image registration and mosaicking to generate a high-precision orthophoto footprint coverages of study area’s sample sites (Figure 3). The resulting orthophoto provides a reliable data foundation for subsequent photovoltaic panel change detection and analysis.

#### 2.2.2. Construction of the Photovoltaic Panel Remote Sensing Change Detection Dataset

To construct the PV panel remote sensing change detection dataset, this study focused on identifying changes in the presence or absence of PV panels within the study area. The post-change imagery was obtained from UAV-based visible light remote sensing data, while the pre-change imagery could not be collected via UAV due to temporal constraints and the unavailability of earlier on-site data. This limitation was addressed by using temporally corresponding, historical visible light imagery of the same region which was obtained from Ovi Earth (Beijing Yuansheng Huanet Technology Co., Ltd., Beijing, China).

The UAV images were geotagged with RTK-based GNSS/IMU information and processed to generate an orthorectified mosaic. Specifically, the UAV imagery was orthorectified and mosaicked in the MS600Pro supporting software (Yusense Map V3.1.0) to produce georeferenced GeoTIFF products. The historical Ovi Earth images were reprojected to the same coordinate reference system as the UAV orthomosaic (e.g., WGS84/UTM) and co-registered to the UAV mosaic using a two-stage strategy: (i) coarse alignment based on geocoordinates, followed by (ii) fine registration using manually selected tie points from stable, non-changing landmarks (e.g., road intersections and building corners). An affine (or low-order polynomial) transformation was estimated from the tie points, and resampling was performed using bilinear interpolation. Registration accuracy was evaluated using independent check points that were not involved in model fitting, and image pairs were retained only when the alignment RMSE was below 1.0 pixel; otherwise, samples with noticeable residual misalignment were excluded to avoid pseudo-changes caused by registration errors [41].

**Figure 4:**
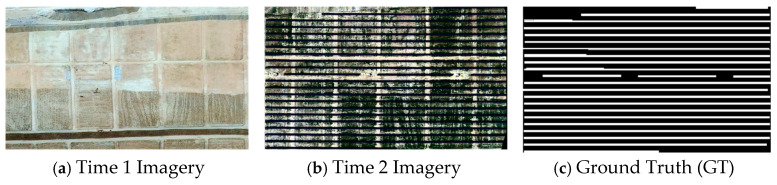
Data samples for PV panel change detection. (**a**) Time 1 Imagery, (**b**) Time 2 Imagery, (**c**) Ground Truth (GT).

To reduce illumination-induced variability, radiometric calibration was conducted for the UAV imagery using a calibrated gray reference panel captured under the same illumination conditions during data acquisition. The panel reflectance values were 0.62 at 450 nm, 0.61 at 555 nm, and 0.61 at 660 nm. After data collection, the built-in radiometric correction workflow in Yusense Map was applied to perform panel-based calibration and radiometric correction, producing reflectance-consistent UAV images for subsequent annotation and dataset construction [42]. Thereafter, ENVI (V5.6) was employed to manually annotate PV panel regions and generate pixel-level change labels, resulting in the final PV panel change detection dataset. Since PV panel boundaries can be ambiguous due to shadowing, mixed pixels, and slight residual misregistration, manual labeling may introduce small uncertainties, mainly around object edges. To reduce inter-annotator variability, the annotations followed a unified guideline and were additionally reviewed to correct ambiguous regions, thereby minimizing potential label noise in the constructed dataset. Figure 4 shows representative samples of the constructed dataset.

## 3. Methods

### 3.1. Architecture Overview

**Figure 5:**
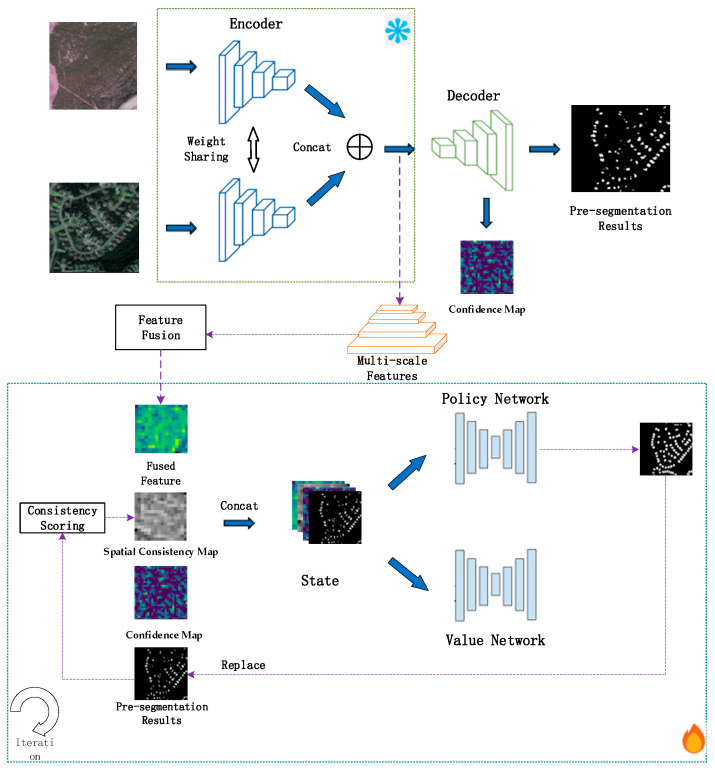
Overall Framework of the Proposed Method. Modules marked with 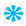 indicate frozen parameters, whereas modules marked with 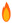 indicate trainable parameters.

Figure 5 shows the proposed network architecture.

As depicted in Figure 5, the proposed change detection architecture takes bi-temporal remote sensing images as input. A shared-weight U-Net network is first pre-trained to achieve competent change detection performance. This pre-training follows the conventional deep learning paradigm: using the same dataset and data split as the subsequent reinforcement learning stage, the network is trained until the validation loss stabilizes and no longer decreases significantly. After pre-training, the encoder parameters are frozen to serve as a feature extractor. The frozen encoder processes the input bi-temporal remote sensing images, generating multi-scale features that encapsulate both low-level spatial details and high-level semantic information of ground objects.

Subsequently, the multi-scale features extracted by the U-Net encoder are fed into a specifically designed multi-scale feature fusion module. This module fuses the multi-scale features derived from the two temporal images to enhance the discriminability of change-related features and suppress irrelevant redundant information. Ultimately, it outputs fused multi-scale features, which serve as the fundamental visual feature input for the subsequent RL branch.

Parallel to the feature extraction process, the pre-trained U-Net outputs an initial binary prediction map, which discriminates between “change” and “no-change” classes. Based on this initial prediction map, two auxiliary cue maps are constructed to guide the RL-based optimization process: one is the confidence map, which is derived from the probability distribution of the U-Net’s output logits to quantify the reliability of the U-Net’s predictive output for each pixel; the other is the spatial consistency map, which is obtained by analyzing the neighborhood similarity of pixels in the initial prediction map to evaluate the spatial continuity of the predicted change regions.

The RL branch serves as the core dynamic optimization component of the proposed architecture. Its state input is formulated by concatenating four components, namely the fused multi-scale features, initial prediction map, confidence map, and spatial consistency map. Under the guidance of pre-defined reward signals, the RL branch iteratively adjusts its optimization policy to refine the initial prediction map. It is noteworthy that the architecture incorporates a dynamic state update mechanism: subsequent to each iteration of optimization, the initial prediction map is updated to the corrected result generated by the RL branch; the confidence map is recalculated based on the updated prediction map via a pseudo-logit construction strategy; and the spatial consistency map is renewed by analyzing the local neighborhood continuity of the updated prediction map. This dynamic update mechanism forms a closed-loop optimization process of “state-action-reward-update” within the RL branch.

Overall, the proposed architecture integrates the superior feature extraction capability of the pre-trained U-Net with the dynamic optimization advantage of RL, forming a two-stage framework. In the first stage, the U-Net is employed to complete preliminary feature extraction and change prediction. In the second stage, the RL branch is leveraged to implement adaptive refinement of the prediction results under the guidance of confidence and spatial consistency cues. The entire architecture achieves an organic integration of static feature learning and dynamic result optimization, laying a solid foundation for enhancing the accuracy and robustness of change detection [43,44,45].

### 3.2. Feature Fusion Module

**Figure 6:**
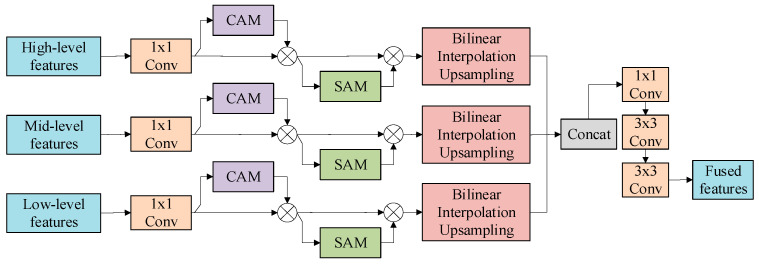
Structure of the fusion module.

The multi-scale feature fusion module, based on an attention mechanism, takes the multi-scale features extracted by the Siamese U-Net encoder as input. It implements a three-step process—channel alignment, attention enhancement, and resolution reconstruction—to fuse and enhance cross-scale features, ultimately outputting a high-resolution feature map rich in semantic contextual information. The fusion module is illustrated in Figure 6.

In the illustration shown above, the module first employs parallel 1D convolutions to unify the channel dimensions of input features from different scales by reducing the semantic gaps across these scales after which, Subsequently, a sequentially structured dual attention mechanism, comprising channel and spatial attention is introduced. The channel attention component calibrates feature importance by leveraging global average pooling followed by a two-layer MLP to learn channel-wise significance which enhances the response of discriminative features. The spatial attention component aggregates features along the channel dimension using both average and max pooling operations. A convolutional layer then processes these aggregated features to generate a spatial weight map, which accentuates change-sensitive regions [46,47,48].

Following attention enhancement, the multi-scale features are upsampled to the target resolution via bilinear interpolation and concatenated along the channel dimension to preserve multi-scale contextual information. Finally, a 1 × 1 convolution followed by two 3 × 3 convolutional layers are applied to fuse and refine the features, strengthening their representational capacity and ensuring local consistency.

### 3.3. Reinforcement Learning-Based Optimization Module Design

#### 3.3.1. State Representation

State representation is a crucial prerequisite for the effective operation of the RL module, as its design quality directly determines whether the RL agent can accurately perceive the current environment and make rational optimization decisions [49].

The state $S_{t}$ of the RL module at each step consists of four core components. The detailed composition of the state space is elaborated as follows:

(1) Fused feature maps

The fused feature maps are derived from the attention mechanism-based multi-scale feature fusion module. As the fundamental visual feature input to the RL branch, these fused feature maps provide essential visual contextual support, laying a solid foundation for the RL network to implement targeted optimization of segmentation results.

(2) Segmentation Prediction Map from the Current Iteration

This component is the core optimization object of the RL policy network. In the first iteration, the segmentation prediction map is the initial binary change map output by the pre-trained U-Net, representing the preliminary judgment of the deep segmentation model on the change areas. In subsequent iterations, this map is updated to the corrected segmentation result output by the RL policy network in the previous iteration, serving as the starting point for the current optimization policy of the RL agent. This component reflects the current “segmentation intent” of the framework and records the continuous attention and adjustment process applied to the target change regions across historical iterations; incorporating this map into the state provides the RL network with guidance on which regions to focus on, thereby avoiding excessive exploration by the RL agent.

(3) Segmentation Error Feedback Map

The confidence map functions as a critical auxiliary cue for quantifying the reliability of the current segmentation predictions. Its generation is intrinsically linked to the model’s logits output during the initial iteration and the dynamic correction behavior of the RL agent in all subsequent iterations [50].

During the first iteration, the confidence map is computed directly from the logits output of the pre-trained U-Net via probability transformation. Let $z_{i,j}\in R^{2}$ denote the logits vector for pixel $\left(i,j\right)$ from the U-Net, with its two dimensions corresponding to the “no-change” (class 1) and “change” (class 2) categories, respectively. The class probability for the pixel is obtained by applying the Softmax function:

(1) $$p_{i,j,k}=Softmax\left(z_{i,j,k}\right)=\frac{\exp\left(z_{i,j,k}\right)}{\sum_{m=1}^{2}\exp\left(z_{i,j,m}\right)},\;k=1,2$$

where $p_{i,j,k}$ (1) is the probability of pixel $\left(i,j\right)$ belonging to class k. The initial confidence score $c_{i,j}^{0}$ (2) (where the superscript 0 denotes the initial iteration) is then defined as the maximum class probability across the two categories:

(2) $$c_{i,j}^{0}=max\{p_{i,j,1},p_{i,j,2}\}$$

Here, $c_{i,j}^{0}\in [0,1]$, with a higher value indicating a more reliable initial prediction for that pixel by the U-Net.

Imaging-driven rationale. In optical remote sensing/UAV orthophotos, the separability of “change vs. no-change” at each pixel is strongly constrained by imaging physics and sensor sampling: (i) spectral similarity between target and background (e.g., desert sand vs. certain PV materials under varying illumination/viewing angles) leads to weak inter-class evidence; (ii) high-frequency texture and repetitive patterns (e.g., PV panel arrays) can cause local feature ambiguity; (iii) boundary mixing caused by finite spatial resolution, point spread function (PSF), orthorectification resampling, and compression introduces mixed pixels and blurred edges; and (iv) illumination/shadow variation and slight misregistration can produce pseudo-changes around high-contrast edges. These effects typically reduce the Softmax separation between the two classes, making the posterior distribution less peaked and thereby lowering the confidence.

To make this link explicit, besides using $c_{i,j}^{0}=\max\left\{p_{i,j,1},p_{i,j,2}\right\}$, one may equivalently interpret uncertainty via the probability margin or entropy:

(3) $$∆p_{i,j}=\left|p_{\left(i,j,2\right)}-p_{\left(i,j,1\right)}\right|$$

(4) $$H_{\left(i,j\right)}=-\sum_{k=1}^{2}p_{\left(i,j,k\right)}logp_{\left(i,j,k\right)}$$

A small $∆p_{i,j}$ (3) (or large $H_{\left(i,j\right)}$ (4)) is frequently observed near object boundaries, weak-texture regions, or spectrally similar areas, which is consistent with the imaging-induced ambiguity described above. In this sense, the confidence map serves as an “imaging-aware reliability proxy” that guides the RL agent to prioritize corrections where the segmentation is most likely affected by resolution limits, texture confusion, or spectral/illumination artifacts.

For subsequent iterations t (where t≥1), the segmentation prediction is the binary mask $M_{i,j}^{t}\in \{0,1\}$ corrected by the RL agent ($M_{i,j}^{t}=1$ for “change”, $M_{i,j}^{t}=0$ for “no-change”). Since this corrected mask lacks inherent logits, we introduce a pseudo-logit construction strategy based on a pixel’s cumulative correction history to generate the confidence map. A core variable $n_{i,j}^{t}$ is defined, representing the cumulative number of times the label for pixel $\left(i,j\right)$ has been modified from the initial iteration up to the current iteration t. Thus, $n_{i,j}^{t}\geq 0$, where $n_{i,j}^{t}=0$ indicates the pixel’s label has never been changed, and $n_{i,j}^{t}\geq 1$ means it has been modified $n_{i,j}^{t}$ times.

The confidence score $c_{i,j}^{t}$ (5) for pixel $\left(i,j\right)$ at iteration t is dynamically adjusted based on $n_{i,j}^{t}$, following the principle that confidence should decrease with an increasing number of modifications until a minimum threshold is reached. This is formalized as:

(5) $$c_{i,j}^{t}=max\{c_{\max}-\Delta c\times n_{i,j}^{t},c_{\min}\}$$

where $c_{\max}=0.9$ represents the maximum confidence assigned to unmodified pixels, Δc=0.2 is the confidence decrement applied per modification, and $c_{\min}=0.3$ is the minimum confidence threshold. The maximum confidence $c_{\max}$ is not set to 1.0 to avoid the reinforcement learning (RL) agent losing its ability to correct initial prediction errors. The selection of this value is based on a statistical analysis of the Softmax probabilities of correctly predicted pixels by the pre-trained model on the validation set, where the mean value is approximately 0.91 and the median is 0.89; thus, $c_{\max}$ is set to 0.9.

The design of the minimum confidence threshold $c_{\min}=0.3$ and the single modification decrement Δc=0.2 is intended to guide the RL agent to focus on key uncertain regions, while avoiding excessive density of confidence values that would lead to an overly large state space. If the distribution of intermediate confidence values is too dense, it will significantly increase the exploration difficulty of the RL agent. This mechanism ensures that initially stable pixels obtain high confidence, and the confidence gradually decays in a controllable and interpretable manner during multiple modifications, ultimately stabilizing around the set minimum threshold [51,52,53].

To generate corresponding pseudo-logits ${\hat{z}}_{i,j}^{t}\in R^{2}$ that are consistent with the score $c_{i,j}^{t}$, we construct them to satisfy the Softmax probability constraint where the probability of the current assigned label equal $c_{i,j}^{t}$:

(6) $${\hat{z}}_{i,j}^{t}=\left\{\begin{array}{c} \left[ln(\frac{1-c_{i,j}^{t}}{c_{i,j}^{t}}),0\right]\;\;\;\;\;\;\;\;\;\;\;\;\;\;\;\;\;\;\;\;\;\;\;if\;M_{i,j}^{t}=1\\ \left[0,ln(\frac{1-c_{i,j}^{t}}{c_{i,j}^{t}})\right]\;\;\;\;\;\;\;\;\;\;\;\;\;\;\;\;\;\;\;\;\;\;\;if\;M_{i,j}^{t}=0 \end{array}\right.$$

Substituting ${\hat{z}}_{i,j}^{t}$ (6) into the Softmax function verifies that $max\left\{\mathrm{Softmax}({\hat{z}}_{i,j}^{t})\right\}=c_{i,j}^{t}$, ensuring consistency. This pseudo-logit construction method directly links the RL agent’s correction history (encapsulated in $n_{i,j}^{t}$) to the confidence map. Consequently, the map dynamically reflects the reliability of the ever-evolving segmentation results and strategically guides the RL agent to focus its optimization efforts on low-confidence pixels—those that have been frequently modified.

Physical meaning of correction-history confidence. The cumulative modification count $n_{i,j}^{t}$ is not only a learning heuristic but also a practical indicator of “persistent ambiguity” under real imaging conditions: pixels repeatedly flipped across iterations tend to concentrate around (a) boundaries blurred by PSF/resampling, (b) thin structures close to the sensor ground sampling distance (GSD), (c) shadow edges that move temporally, and (d) misregistration-induced double-edge patterns. Therefore, decreasing confidence with increasing $n_{i,j}^{t}$ can be justified as an imaging-consistent rule: repeated corrections imply that the local evidence is unstable under the resolution/noise/texture constraints, so the RL agent should treat such pixels as low-reliability and rely more on neighborhood structure and fused multi-scale features to resolve them.

(4) Spatial Consistency Map

The spatial consistency map is designed based on the spatial distribution characteristics of ground objects in remote sensing images (i.e., authentic change regions are typically spatially contiguous). It evaluates the spatial continuity of the change regions in the current segmentation prediction map by analyzing the neighborhood similarity of pixels.

In both the first and subsequent iterations, the spatial consistency map is calculated using a 3 × 3 convolutional kernel: the convolutional kernel slides over the current segmentation prediction map to count the number of pixels with the same segmentation label as the central pixel in the 3 × 3 neighborhood. The count value is then normalized by the total number of pixels in the neighborhood (i.e., 9) to obtain the spatial consistency score of each pixel. A higher score indicates that the pixel’s segmentation label is more consistent with its neighboring pixels, meaning the region is more likely to be an authentic change or no-change region. By incorporating this map into the state space, the RL agent can identify isolated erroneous pixels (with low spatial consistency scores) that violate the spatial laws of ground objects and perform targeted correction.

Let $N_{\left(i,j\right)}=\left\{\left(u,v\right)|u\in \left[i-1,i,i+1,v\in \left[j-1,j,j+1\right]\right]\right\}$ denote the 3 × 3 neighborhood and $M_{\left(i,j\right)}^{t}\in \left\{0,1\right\}$ be the current binary mask. The spatial consistency score is defined as:

(7) $$S_{\left(i,j\right)}^{t}=\frac{1}{\left|N_{\left(i,j\right)}\right|}\sum_{(u,v)\in N_{\left(i,j\right)}}1(M_{\left(u,v\right)}^{t}=M_{\left(i,j\right)}^{t})$$

where 1(·) is the indicator function and $\left|N_{\left(i,j\right)}\right|=9$ (border pixels handled by padding or valid neighborhood size).

Imaging-driven rationale and relation to texture/boundaries/artifacts. Under finite spatial resolution and PSF blur, truly changed objects in optical remote sensing typically occupy multiple adjacent pixels and form connected components at a scale larger than random noise; conversely, isolated single-pixel changes are often caused by sensor noise, quantization/compression artifacts, orthorectification resampling, or slight misregistration (especially around high-contrast edges). Thus, $S_{\left(i,j\right)}^{t}$ (7) acts as a local “contiguity prior” consistent with imaging formation and sampling, suppressing pepper-noise-like false alarms and promoting region-level continuity.

Importantly, boundaries are a special case: genuine object edges naturally have mixed neighborhoods (lower $S_{\left(i,j\right)}^{t}$(7)), particularly when the boundary width is close to the GSD or when texture is highly anisotropic (e.g., PV panel grid patterns). Therefore, the spatial consistency map is not used alone; it is jointly interpreted with (i) the fused feature maps (providing texture/edge evidence) and (ii) the confidence map (capturing spectral/illumination ambiguity). This joint state allows the RL policy to distinguish “low-consistency due to true boundaries” from “low-consistency due to imaging artifacts,” preserving sharp edges when supported by feature evidence while removing isolated artifacts when confidence is low and neighborhood support is absent.

In summary, the state space integrates multi-dimensional information including visual features, segmentation results, prediction reliability, and spatial distribution laws. This comprehensive state representation enables the RL agent to fully perceive the current optimization status, thereby making more accurate and efficient correction decisions. The four components of the state space are concatenated along the channel dimension to form a unified input tensor for the RL policy network, ensuring the compatibility and integrity of the input information.

#### 3.3.2. Reward Function

To effectively guide the RL policy network in optimizing the initial semantic segmentation results, this paper designs a reward function based on the performance improvement in change detection. The core objective of the reward function is to measure the degree of overall performance enhancement in the segmentation result after each policy adjustment compared to the original prediction, thereby incentivizing the policy network to progressively correct misclassified areas.

The proposed reward function comprehensively considers two metrics: the improvement in mean Intersection over Union (mIoU) and the improvement in mean F1-score (mF1):

ΔmIoU: Measures the increase in the average Intersection over Union between the prediction and the ground truth label before and after the policy optimization.

ΔmF1: Measures the improvement in overall prediction accuracy, balancing both precision and recall.

The final immediate reward $r_{t}$ at step t is defined as follows:

(8) $$r_{t}=\left(m{IoU}_{after}-m{IoU}_{before}\right)+\left(m{F1}_{after}-m{F1}_{before}\right)$$

where:

$m{IoU}_{after}$, $m{IoU}_{before}$ represent the mIoU before and after optimization, respectively.

$m{F1}_{after}$, $m{F1}_{before}$ represent the mF1-score before and after optimization, respectively.

To account for the impact of each policy adjustment on subsequent segmentation performance, the discounted cumulative reward $R_{t}$ is introduced:

(9) $$R_{t}=r_{t}+\gamma r_{t+1}+\gamma^{2}r_{t+2}+\ldots +\gamma^{T-t}r_{T}$$

where:

γ∈[0,1] is the discount factor, determining the present value of future rewards.

T is the total number of steps in the entire optimization process.

#### 3.3.3. Reinforcement Learning Training Mechanism

**Figure 7:**
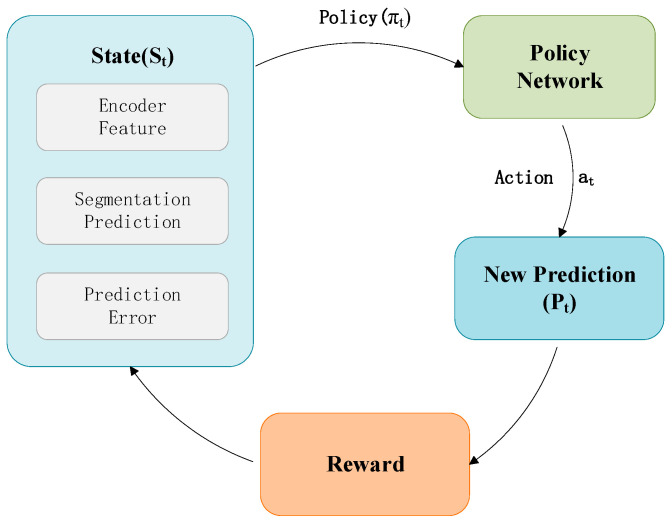
Reinforcement Learning Training Process.

In this study, to optimize the initial segmentation results and achieve fine-grained adjustment of change regions, the RL module employs the Proximal Policy Optimization (PPO) algorithm for training, with the overall procedure being illustrated in Figure 7 below. As an efficient and stable policy gradient method, PPO introduces a clipping mechanism during policy updates. This effectively mitigates training instability caused by drastic shifts in the policy distribution, making it particularly suitable for the present task characterized by a complex state space, sparse feedback signals, and an optimization process requiring multi-iteration adjustments [54]. The RL training mechanism is illustrated in Figure 7.

At each iteration, the RL module receives the state $s_{t}$. Based on this input, the policy network (Actor) outputs a pixel-level action $a_{t}$, which performs fine-grained adjustments to the current segmentation probabilities according to:

(10) $$p_{i}\left(t+1\right)=clip\left(p_{i}\left(t\right)+d_{i}\left(t\right)\right),\;i=1,\ldots ,N$$

where $p_{i}\left(t\right)$(10) is the segmentation probability of the i-th pixel, $d_{i}\left(t\right)$ is the adjustment value output by the policy network, and the clip function constrains the probability to the interval [0,1].

The Actor-Critic architecture within the PPO framework consists of:

Policy Network (Actor): Learns a parameterized policy $\pi_{\theta }(a$|s) to generate pixel-level actions.

Value Network (Critic): Estimates the state value $V_{\phi }\left(s\right),$ to assist in computing the advantage function A(s,a):

(11) $$A\left(s_{t},a_{t}\right)=R_{t}-V_{\phi }\left(s_{t}\right)$$

where $R_{t}$(9) denotes the cumulative reward.

The policy network is updated based on the clipped probability ratio, with its policy loss defined as:

(12) $$L_{policy}\left(\theta \right)=-E_{t}\left[\min\left(r_{t}\left(\theta \right)A\left(s_{t},a_{t}\right),clip\left(r_{t}\left(\theta \right),1-\epsilon ,1+\epsilon \right)A\left(s_{t},a_{t}\right)\right)\right]$$

The value network is updated by minimizing the mean squared error between the predicted state value and the cumulative reward:

(13) $$L_{value}\left(\phi \right)=\frac{1}{2}\sum_{t}{\left(R_{t}-V_{\phi }\left(S_{t}\right)\right)}^{2}$$

Furthermore, to prevent the policy from collapsing to a deterministic output and to encourage exploration, an entropy regularization term $H[\pi_{\theta }]$ for the policy is incorporated during training. The final combined loss function is:

(14) $$L=L_{policy}+L_{value}-\beta H[\pi_{\theta }]$$

The reward function is defined based on the improvement in segmentation performance, jointly considering the mIoU and mF1 metrics: a positive reward is given if the result improves over the previous prediction, while a negative reward is assigned upon degradation, thereby guiding the policy towards better performance.

The RL process employs multi-episode iterative optimization. In each episode, the policy network continuously refines the segmentation probabilities until convergence. To ensure training efficiency and prevent overfitting, a dynamic termination mechanism is implemented: an episode concludes automatically when either the maximum number of iterations is reached, or the improvement in performance over two consecutive rounds falls below a predefined threshold [55].

## 4. Results and Discussion

### 4.1. Experimental Setup

#### 4.1.1. Implementation Details

During the experiments, all input multi-temporal remote sensing images were resized to 256 × 256 patches before being fed into the network to meet model input requirements and reduce computational overhead. The prediction results were restored to the original image resolution during the inference stage for final performance evaluation.

Regarding the training pipeline, the deep learning branch first generated an initial change detection result based on the Siamese U-Net. This initial segmentation map was then used as one of the inputs to the RL module. The RL component was trained using the PPO algorithm, with its training and testing sets strictly aligned with the previously defined partitions of the CDD, SYSU-CD, and PVCD datasets.

For the action space design, a discrete action set was defined as:

A={±0.1,±0.2,±0.5}

where each action represents an increment or decrement applied to the current pixel’s segmentation probability. The final output probability is constrained to the interval [0, 1] using a clip function to ensure numerical stability.

It is worth noting that the PPO algorithm, as a policy gradient–based RL method, is inherently insensitive to the absolute magnitude of discrete action values. The optimization direction of the policy is primarily determined by the advantage function, which evaluates the relative benefit of each action under the current state. Therefore, as long as the action value range remains within a reasonable scale—avoiding excessively large values that cause oscillation and overly small values that slow convergence—the policy network can autonomously learn the appropriate action selection strategy for different regions. This property allows the proposed framework to maintain adaptability and stability while performing pixel-level probabilistic refinements.

In terms of hyperparameter settings, PPO training was conducted in an online manner with the batch size fixed at 1, optimizing one image pair step-by-step per iteration. The learning rate was initialized to 0.001 and gradually decayed during training. The Adam optimizer was employed, with the discount factor γ=0.95, the PPO clipping parameter ϵ=0.2, and the entropy regularization coefficient β=0.01 to encourage exploration and prevent premature policy collapse. All RL experiments were conducted with fixed random seeds (Python V3.8/NumPy V1.24.4/PyTorch V1.13.0), using seeds {0, 1, 2}. The PPO Actor–Critic network contains 2,677,352 parameters in total (shared encoder: 675,521; Actor head: 690,086; Critic head: 1,311,745). The exact model configuration is provided in Appendix A.

For the training iterations, the maximum training steps were set to 60,000 for the SYSU-CD dataset, 40,000 for the CDD dataset, and 30,000 for the PVCD dataset.

Furthermore, to prevent overfitting, a dynamic early stopping mechanism was introduced during the experiments: training was automatically terminated if the improvement on the validation set (measured by mIoU and F1-score) remained below ∆=0.01 for K=5 consecutive evaluations. The evaluation metrics employed were mIoU and the F1-score, providing a comprehensive assessment of the model’s performance in terms of both pixel-level accuracy and overall consistency in identifying change regions.

#### 4.1.2. Comparative Experiments on Segmentation Methods

To evaluate the effectiveness of the proposed reinforcement learning (RL)-enhanced framework, we conducted systematic experiments on three public datasets (CDD, SYSU-CD, and BRIGHT) as well as our self-constructed PVCD. In addition, to further verify the generalization and plug-and-play capability of the RL module, we applied the same RL optimization strategy to three representative deep change detection networks, including SNUNet-CD and two Transformer-based models (BIT and ChangeFormer). The main objective of these experiments is to examine whether the RL mechanism can consistently refine initial segmentation results across different network architectures and under different data distributions and imaging conditions, rather than being effective only for a single backbone.

As shown in Table 2, integrating the proposed RL refinement consistently improves the corresponding pretrained baselines across key evaluation metrics on all four datasets (CDD, SYSU-CD, PVCD, and BRIGHT). Specifically, using SiamU-Net as an example, the RL-enhanced model improves mIoU by 3.07 points on CDD, 2.54 points on SYSU-CD (with more complex scenes and diverse change patterns), 6.13 points on PVCD, and 3.1 points on BRIGHT. Moreover, the same RL module yields consistent performance gains when combined with other representative change detection backbones, with mIoU and F1-score generally improving across datasets. These results indicate that the proposed RL mechanism provides robust adaptability and iterative refinement capability under diverse image characteristics and change scenarios.

**Figure 8:**
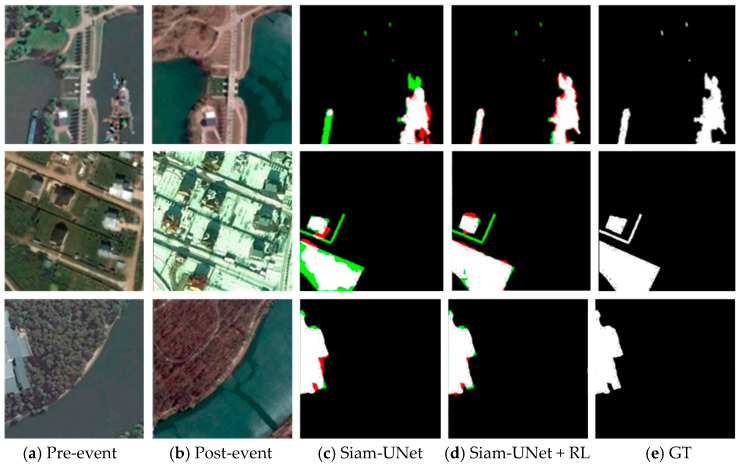
Visualization results on the CDD dataset (spatial resolution: 0.03–1.0 m). (**a**) Pre-event image; (**b**) Post-event image; (**c**) SiamU-Net prediction; (**d**) SiamU-Net + RL refinement; (**e**) GT. False positives are highlighted in red, and false negatives are indicated in green.

**Figure 9:**
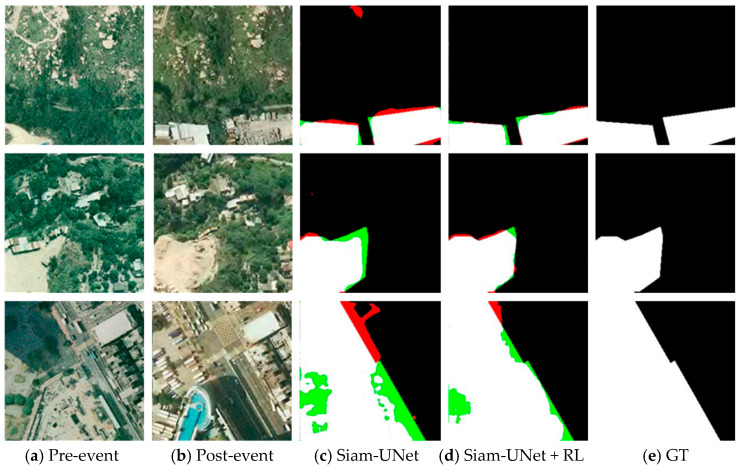
Visualization results on the SYSU-CD dataset (spatial resolution: 0.5 m GSD). (**a**) Pre-event image; (**b**) Post-event image; (**c**) SiamU-Net prediction; (**d**) SiamU-Net + RL refinement; (**e**) GT.

**Figure 10:**
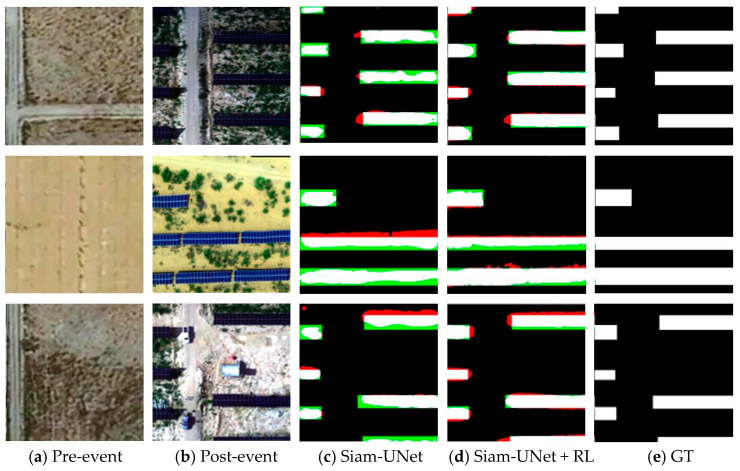
Visual depictions of the results from the PVCD dataset. (spatial resolution: 1 m GSD). (**a**) Pre-event image; (**b**) Post-event image; (**c**) SiamU-Net prediction; (**d**) SiamU-Net + RL refinement; (**e**) GT.

**Figure 11:**
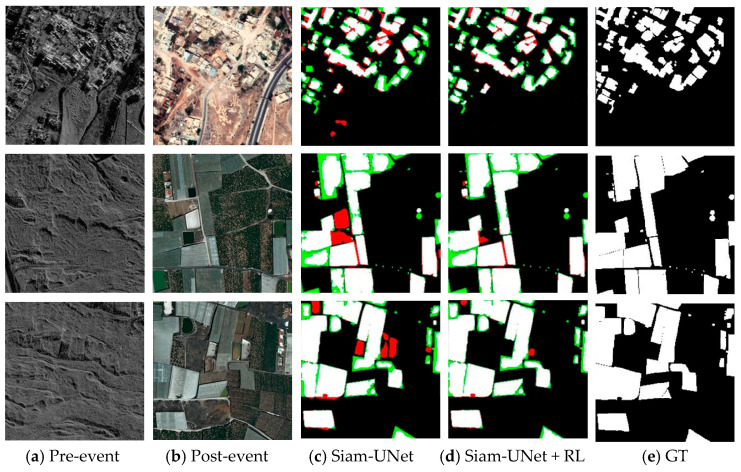
Visual depictions of the results from the BRIGHT dataset (optical imagery; spatial resolution: 0.3–1 m). (**a**) Pre-event image; (**b**) Post-event image; (**c**) SiamU-Net prediction; (**d**) SiamU-Net + RL refinement; (**e**) GT.

As illustrated in Figure 8, Figure 9, Figure 10 and Figure 11, the visual results provide intuitive evidence of the improvements achieved by RL. In representative scenes, the initial predictions produced by the pretrained backbone often suffer from blurred boundaries, fragmented regions, and local omissions. After RL-based refinement, these errors are reduced, yielding outputs with improved spatial continuity, more complete object shapes, and sharper boundaries that better match the ground truth. Notably, the larger improvement on PVCD can be attributed to the acquired imagery, which typically has higher imaging quality and stronger spatial coherence, making the spatial-consistency–guided RL refinement more effective.

From an imaging perspective, these gains stem from how the RL agent leverages confidence and spatial-consistency cues to mitigate common remote-sensing uncertainties. Boundary blur is often related to limited spatial resolution and mixed pixels along object contours, where the evidence is unstable and confidence tends to be low; RL therefore performs multi-step, small-amplitude probability corrections in these uncertain boundary regions to improve boundary fidelity. Meanwhile, shadow/illumination differences, radiometric inconsistencies, orthorectification resampling artifacts, and residual misregistration can induce isolated or weakly connected pseudo-change responses. By incorporating a spatial consistency map, RL suppresses such locally incoherent artifacts while preserving true boundaries where neighborhood agreement naturally drops. Overall, RL acts as a feedback-driven, adaptive interpretation constraint that refines the posterior change map, sharpening boundaries. Zoomed-in boundary insets for visual verification are provided in the Supplementary Materials (Figure S2).

In summary, the experimental results comprehensively validate the effectiveness of the proposed RL optimization mechanism. The method not only achieves consistent improvements in quantitative metrics but also delivers superior qualitative performance, exhibiting stronger semantic consistency and regional integrity in the visualized results. These findings confirm its practical value and generalization capability in remote sensing change detection tasks.

After introducing RL, the computational metrics of the network are shown in Table 3.

### 4.2. Validation

#### 4.2.1. Validation of State Space Design

To validate the rationale behind the state space design in our proposed RL framework, systematic ablation studies were conducted across four datasets.

Regarding feature fusion, we configured different levels of feature combination:

- **Single-Scale (High-Level)**: Utilizing only the highest-level features.
- **Two-Scale Fusion**: Fusing features from the last two levels.
- **Three-Scale Fusion**: Fusing features from the last three levels.
- **Four-Scale Fusion**: Fusing features from all four levels.

Experimental results indicated that the model employing three-scale fusion achieved the best performance across all metrics on both datasets, while incorporating all four levels did not yield significant further improvement. This suggests that a moderate degree of multi-scale fusion in state modeling achieves an optimal balance between performance and computational efficiency.

For the validation of state components, we adopted the best-performing Three-Scale Fusion model as the baseline and conducted controlled experiments by individually removing three key components from the state space, namely the initial prediction map, confidence map, and spatial consistency map:

- ***w/o* Initial Prediction Map:** Removing the initial prediction map to examine the role of preliminary segmentation judgment information in guiding the RL agent’s optimization process.
- ***w/o* Confidence Map:** Removing the confidence map to investigate the impact of prediction reliability information on the optimization performance of the RL agent.
- ***w/o* Spatial Consistency Map:** Removing the spatial consistency map to evaluate the significance of spatial distribution prior information for guiding accurate change region correction.

Table 4 shows the results of ablation analysis.

Table 4 results show that removing any single component causes a significant performance drop, confirming the indispensability of all three in state representation. Notably, removing the initial prediction map leads to the most pronounced performance degradation, further verifying its critical role.

In summary, the ablation study systematically validates the effectiveness of our state space design. The results demonstrate that: in terms of feature fusion, integrating the last three encoder levels (conv3, conv4, conv5) optimally balances semantic information and spatial details, which boosts the proposed method’s performance by providing usable environmental details. Regarding component composition, the multi-scale fused features establish the foundation for global perception, the initial prediction map ensures the coherence of iterative optimization, the confidence map guarantees the targeting of optimization by highlighting uncertain regions, and the spatial consistency map aids in distinguishing authentic change regions based on spatial prior knowledge. These components work synergistically to form the critical informational foundation that drives the agent towards efficient pixel-level optimization.

#### 4.2.2. Sensitivity Analysis of Hyperparameters

To investigate the sensitivity of the proposed framework to key hyperparameters, we conducted experiments on four benchmark change detection datasets (CDD, SYSU-CD, PVCD, BRIGHT). The hyperparameter examined here is the pixel-level action set A in PPO.

We defined three discrete action sets:

- A1: {±0.05, ±0.1, ±0.2}
- A2: {±0.1, ±0.2, ±0.5}
- A3: {±0.2, ±0.5, ±1.0}

Each action represents an increment or decrement applied to the current pixel’s segmentation probability. Experimental results show that the moderate action set A2 consistently achieves the best balance between segmentation accuracy (mIoU and F1-score) and training efficiency across all datasets. Very small steps (A1) lead to slower convergence, whereas overly large steps (A3) reduce accuracy due to overshooting. Table 5 shows the results of the sensitivity analysis for different action sets across the three datasets.

The sensitivity analysis demonstrates that the pixel-level action set significantly affects both segmentation accuracy and convergence. Across all three datasets (CDD, SYSU-CD, PVCD, BRIGHT), the moderate action set A2 {±0.1, ±0.2, ±0.5} consistently achieves the best balance, yielding the highest mIoU and F1 while requiring fewer convergence iterations. Small steps (A1) result in slower convergence, whereas large steps (A3) reduce accuracy due to overshooting. These results indicate that appropriately sized action increments are crucial for effective pixel-level refinement, and A2 is the most suitable choice for robust and efficient change detection across different datasets.

#### 4.2.3. Limitations Analysis

**Figure 12:**
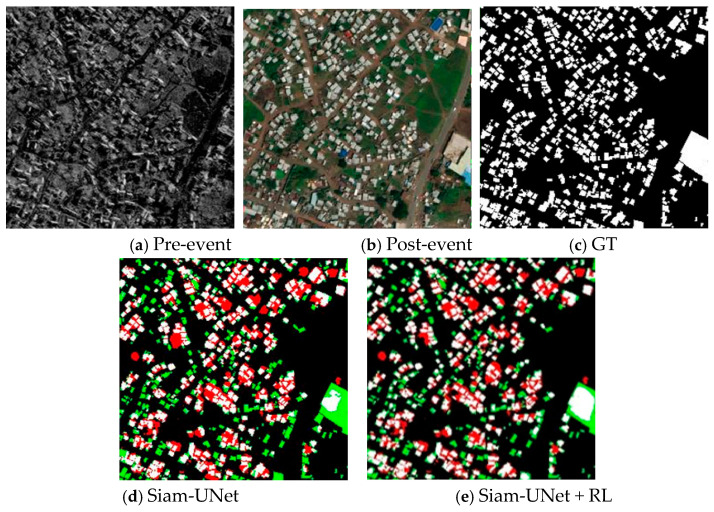
Representative failure cases. (**a**) Pre-event image; (**b**) Post-event image; (**c**) Ground truth (GT); (**d**) SiamU-Net prediction; (**e**) SiamU-Net + RL refinement.

Although the proposed RL-enhanced framework delivers consistent improvements in most scenarios, noticeable performance degradation can still occur under certain challenging conditions. Figure 12 illustrates representative failure cases where change targets are extremely small, highly numerous, and densely distributed, often accompanied by complex local textures or high-frequency background patterns. In such cases, the model is more prone to missing a large portion of micro-target changes, producing fragmented “salt-and-pepper” predictions, and confusing boundaries due to adhesion between adjacent targets. Even after RL-based refinement, the overall gain becomes substantially smaller, and local errors may persist, including over-suppression of true micro-changes or blurred delineation. This limitation mainly stems from the fact that, when targets are too small and densely packed, the initial segmentation evidence and confidence estimates become less stable, while neighborhood-consistency cues become less discriminative because true changes themselves no longer form clearly coherent regions. As the RL module essentially performs iterative corrections on the initial probability map, its refinement ability is constrained by the quality of the initial prediction and the separability of local patterns, making it difficult to recover changes that are largely missed at the first stage and sometimes leading to conservative smoothing. From an application perspective, such scenes should be treated as high-uncertainty cases, where additional verification (e.g., higher-resolution imagery or manual inspection) may be required to mitigate the risk of omissions. Future work may improve robustness in this regime by enhancing small-target feature preservation and representation, introducing priors tailored to dense micro-change patterns, and designing state representations and action policies that better fit densely distributed tiny objects.

## 5. Conclusions

This paper presents an RL-enhanced remote sensing change detection framework that integrates deep learning with reinforcement learning to mitigate limitations of static one-pass inference, particularly for boundary refinement and pseudo-change suppression. The proposed approach couples a change detection backbone with a PPO-based RL module that performs feedback-driven, multi-step probabilistic refinement on the initial change probability map, enabling adaptive correction of local errors beyond fixed-rule post-processing.

Comprehensive experiments on three public benchmarks (CDD, SYSU-CD, and BRIGHT) and our self-constructed PVCD dataset demonstrate that the proposed RL refinement yields consistent gains across datasets and network architectures. Using SiamU-Net as an example, the RL-enhanced model improves mIoU by 3.07 points on CDD, 2.54 points on SYSU-CD, 6.13 points on PVCD, and 3.1 points on BRIGHT; similar improvements are also observed when the RL module is integrated with other representative backbones. Qualitative results further show reduced fragmentation and local omissions, producing change maps with improved spatial continuity, shape integrity, and boundary clarity.

From an imaging and interpretation perspective, these improvements arise from the RL agent’s use of confidence/uncertainty and spatial-consistency cues to mitigate common remote-sensing uncertainties. Low-confidence boundary pixels—often associated with limited spatial resolution and mixed pixels—are refined through iterative, small-step corrections to enhance boundary fidelity. Meanwhile, shadow/illumination variability, radiometric inconsistencies, orthorectification resampling artifacts, and residual misregistration may induce isolated or weakly connected pseudo-changes; incorporating spatial-consistency cues helps suppress such incoherent responses while preserving true boundaries where neighborhood agreement naturally decreases. Overall, the RL module acts as a feedback-driven, adaptive interpretation constraint that sharpens boundaries and reduces artifact-driven false alarms.

Despite the promising results, several limitations remain. Refinement effectiveness can be influenced by sensor-dependent imaging characteristics (e.g., GSD, spectral configuration, noise level, and preprocessing), which affect confidence estimation and local coherence. Moreover, performance may degrade when change targets are extremely small and densely distributed over complex high-frequency textures, where initial evidence becomes unstable and neighborhood consistency is less discriminative; consequently, RL may yield smaller gains or over-suppress micro-changes. Since the RL module iteratively corrects the initial probability map, its capability is constrained by initial prediction quality and local separability. Future work will improve efficiency (e.g., adaptive stopping and lightweight policies), strengthen robustness for dense micro-change scenarios, and extend the current RGB setting to incorporate multispectral cues and sensor-related constraints for broader applicability.

In summary, this study demonstrates that reinforcement learning can serve as an effective, imaging-aware refinement mechanism for remote sensing change detection, improving interpretation quality (boundary fidelity and spatial coherence) alongside accuracy, while highlighting challenging conditions that warrant further investigation.

## Figures and Tables

**Table 1:** Technical specifications of the DJI Matrice 300 RTK UAV.

Parameters	Numerical Value
Volumetric	810 × 670 × 430 mm
Max Take-off Weight	9 kg
Max Payload Capacity	2.7 kg
Max Flight Time (No Payload)	55 min
Max Level Flight Speed	23 m/s (82.8 km/h)
Max Wind Resistance	15 m/s
Hover Accuracy (With RTK)	Vertical: ±0.1 m; Horizontal: ±0.1 m
GNSS Systems	GPS + GLONASS + BeiDou + Galileo
IP Rating	IP45

**Table 2:** Segmentation accuracies of the models on the two datasets.

Methods	CDD			SYSU-CD			PVCD			BRIGHT		
	OA	mIoU	F1	OA	mIoU	F1	OA	mIoU	F1	OA	mIoU	F1
SiamU-Net	97.46 ± 0.06	88.45 ± 0.22	93.64 ± 0.14	88.92 ± 0.10	72.68 ± 0.38	83.67 ± 0.26	82.65 ± 0.16	66.71 ± 0.62	79.49 ± 0.44	95.10 ± 0.09	62.45 ± 0.60	70.05 ± 0.45
SNUNet-CD	97.68 ± 0.05	90.12 ± 0.20	94.01 ± 0.12	89.46 ± 0.10	74.32 ± 0.35	84.05 ± 0.24	84.55 ± 0.14	68.13 ± 0.58	82.23 ± 0.40	95.35 ± 0.09	63.70 ± 0.58	70.90 ± 0.43
BIT	97.55 ± 0.05	90.64 ± 0.18	94.39 ± 0.11	89.87 ± 0.09	73.98 ± 0.34	85.31 ± 0.22	84.36 ± 0.14	68.76 ± 0.56	81.98 ± 0.39	95.28 ± 0.09	64.25 ± 0.56	71.40 ± 0.41
ChangeFormer	98.02 ± 0.04	90.98 ± 0.17	95.02 ± 0.10	89.79 ± 0.09	74.78 ± 0.33	84.61 ± 0.22	85.48 ± 0.12	69.32 ± 0.54	82.15 ± 0.38	95.45 ± 0.08	64.85 ± 0.55	71.85 ± 0.40
SiamU-Net + RL	97.65 ± 0.05	91.52 ± 0.18	95.29 ± 0.11	89.91 ± 0.09	75.22 ± 0.31	85.26 ± 0.21	85.41 ± 0.13	72.84 ± 0.48	84.10 ± 0.34	95.55 ± 0.08	65.55 ± 0.50	72.10 ± 0.36
SNUNet-CD + RL	97.88 ± 0.04	91.60 ± 0.17	95.00 ± 0.10	89.95 ± 0.09	75.85 ± 0.30	85.45 ± 0.20	86.30 ± 0.12	72.10 ± 0.50	84.60 ± 0.35	96.10 ± 0.07	66.70 ± 0.48	72.80 ± 0.35
BIT + RL	97.78 ± 0.04	91.90 ± 0.16	95.10 ± 0.10	90.10 ± 0.08	75.10 ± 0.31	85.95 ± 0.20	86.20 ± 0.12	72.45 ± 0.49	84.90 ± 0.34	95.60 ± 0.08	67.30 ± 0.46	73.20 ± 0.34
ChangeFormer + RL	98.10 ± 0.03	92.05 ± 0.15	95.60 ± 0.09	90.15 ± 0.08	76.05 ± 0.29	86.05 ± 0.19	86.70 ± 0.11	73.00 ± 0.47	85.20 ± 0.33	95.75 ± 0.07	67.80 ± 0.44	73.60 ± 0.33
ChangeMamba	98.06 ± 0.04	91.25 ± 0.17	95.35 ± 0.10	90.05 ± 0.09	75.30 ± 0.32	85.70 ± 0.21	85.95 ± 0.13	70.10 ± 0.53	83.05 ± 0.37	95.50 ± 0.08	65.20 ± 0.54	72.20 ± 0.39

**Table 3:** Comparison of computational performance between networks.

Model	Average Inference Time (ms)	Inference Speed (FPS)	FLOPs (G)	Peak GPU Memory (Train, BS = 32) (GB)	Peak GPU Memory (Infer, BS = 1) (GB)	Total Training Time (h)	Convergence (Best Val. mIoU)	RL Iterations at Inference (K)
SiamU-Net	5.34	187.10	10.66	16.8	1.4	28.3	Trained for 1000 epochs; best at epoch 965	0
SiamU-Net + RL	12.40	80.68	17.50	22.6	2.4	84.6	RL fine-tuned for 2000 epochs; best at epoch 1989 (baseline pretrained for 1000 epochs)	7

**Table 4:** Ablation Results.

Methods	CDD			SYSU-CD			PVCD			BRIGHT		
	OA	mIoU	F1	OA	mIoU	F1	OA	mIoU	F1	OA	mIoU	F1
Single-Scale	95.21	82.37	90.02	85.64	68.95	81.12	81.14	66.57	79.96	94.72	62.42	69.05
Two-Scale Fusion	96.88	86.45	92.51	87.93	72.18	83.49	83.43	69.80	82.33	95.12	64.05	70.85
Three-Scale Fusion	97.65	91.52	95.29	89.91	75.22	85.26	85.41	72.84	84.10	95.55	65.55	72.10
Four-Scale	97.61	89.38	94.15	89.87	75.10	85.38	85.37	72.72	84.02	95.48	65.35	72.33
*w/o* Prediction	93.21	81.58	89.72	84.74	67.25	80.12	80.24	64.87	78.96	93.25	58.90	66.30
*w/o* Confidence	96.52	85.46	92.12	86.35	72.96	82.43	84.47	67.28	81.25	94.95	63.45	70.15
*w/o* Spatial Consistency	97.28	86.95	91.28	88.51	70.18	81.38	84.01	67.80	80.22	94.82	62.80	69.40

**Table 5:** Sensitivity Analysis of Action Set on Four Datasets.

Dataset	Action Set	mIoU (%)	F1 (%)	Convergence Iterations(×10^3^)
CDD	A1 {±0.05, ±0.1, ±0.2}	88.98	93.53	8100
	A2 {±0.1, ±0.2, ±0.5}	91.52	95.29	7750
	A3 {±0.2, ±0.5, ±1.0}	87.13	91.26	7940
SYSU-CD	A1 {±0.05, ±0.1, ±0.2}	73.65	83.83	9720
	A2 {±0.1, ±0.2, ±0.5}	75.22	85.26	9356
	A3 {±0.2, ±0.5, ±1.0}	71.27	82.68	9528
PVCD	A1 {±0.05, ±0.1, ±0.2}	70.81	82.25	8910
	A2 {±0.1, ±0.2, ±0.5}	72.84	84.10	8525
	A3 {±0.2, ±0.5, ±1.0}	68.99	81.75	8734
BRIGHT	A1 {±0.05, ±0.1, ±0.2}	66.92	72.98	9980
	A2 {±0.1, ±0.2, ±0.5}	68.10	74.40	9540
	A3 {±0.2, ±0.5, ±1.0}	65.78	72.10	9720

## Data Availability

The UAV remote sensing data collected and analyzed in this study involve photovoltaic facilities operated by relevant enterprises and therefore cannot be publicly shared due to privacy and confidentiality restrictions. Researchers interested in accessing the PVCD dataset for non-commercial academic research may contact the corresponding author upon reasonable request. A request should include (i) the applicant’s name, affiliation, and institutional email, (ii) a brief research plan and intended use, and (iii) a data management and security statement (e.g., secure storage, restricted access). Access may require signing a Data Use Agreement (DUA)/NDA, and the dataset must not be redistributed to third parties. Approved users are required to cite this paper and acknowledge the data source. To support reproducibility, the model implementation code has been released as open source at: https://github.com/zyl-mwy/RLCD (accessed on 10 January 2026).

## Supplementary Materials

The following supporting information can be downloaded at https://www.mdpi.com/article/10.3390/jimaging12030139/s1: Figure S1: Qualitative comparison of RL refinement under RGB-only and RGB+NIR inputs on the PVCD pilot subset; Table S1: Pilot comparison of RGB-only vs. RGB+NIR on a PVCD subset; Figure S2: High-Resolution Mosaics and Zoom-in Boundary Verification; Table S2: RL gains stratified by dataset type and resolution/GSD proxy.

## Abbreviations

The following abbreviations are used in this manuscript:

CD	Change Detection
RL	Reinforcement Learning
UAV	Unmanned Aerial Vehicle
GT	Ground truth
PV	Photovoltaic
GSD	Ground Sampling Distance

## Appendix A

Table A1 lists the network architecture and specific parameters of SiamU-Net.

**Table A1:** Network architecture and specific parameters of SiamU-Net.

Module	Layer (Type)	Output Shape	Number of Parameters
Input	InputLayer	(−1, 3, 256, 256)	0
Encoder Stage 1	Conv2d (3 → 16, 3 × 3)	(−1, 16, 256, 256)	448
Encoder Stage 2	Conv2d (16 → 16, 3 × 3)	(−1, 16, 256, 256)	2320
	Conv2d (16 → 32, 3 × 3)	(−1, 32, 128, 128)	4640
	Conv2d (32 → 32, 3 × 3)	(−1, 32, 128, 128)	9248
Encoder Stage 3	Conv2d (32 → 64, 3 × 3)	(−1, 64, 64, 64)	18,496
	Conv2d (64 → 64, 3 × 3)	(−1, 64, 64, 64)	36,928
	Conv2d (64 → 64, 3 × 3)	(−1, 64, 64, 64)	36,928
Encoder Stage 4	Conv2d (64 → 128, 3 × 3)	(−1, 128, 32, 32)	73,856
	Conv2d (128 → 128, 3 × 3)	(−1, 128, 32, 32)	147,584
	Conv2d (128 → 128, 3 × 3)	(−1, 128, 32, 32)	147,584
Bottleneck	ConvTranspose2d (128 → 128, stride = 2)	(−1, 128, 64, 64)	147,584
Decoder Stage 4d	ConvTranspose2d (384 → 128, 3 × 3)	(−1, 128, 64, 64)	442,496
	ConvTranspose2d (128 → 128, 3 × 3)	(−1, 128, 64, 64)	147,584
	ConvTranspose2d (128 → 64, 3 × 3)	(−1, 64, 64, 64)	73,792
Decoder Stage 3d	ConvTranspose2d (192 → 64, 3 × 3)	(−1, 64, 128, 128)	110,656
	ConvTranspose2d (64 → 64, 3 × 3)	(−1, 64, 128, 128)	36,928
	ConvTranspose2d (64 → 32, 3 × 3)	(−1, 32, 128, 128)	18,464
Decoder Stage 2d	ConvTranspose2d (96 → 32, 3 × 3)	(−1, 32, 256, 256)	27,680
	ConvTranspose2d (32 → 16, 3 × 3)	(−1, 16, 256, 256)	4624
Decoder Stage 1d	ConvTranspose2d (48 → 16, 3 × 3)	(−1, 16, 256, 256)	6928
	ConvTranspose2d (16 → 1, 3 × 3)	(−1, 1, 256, 256)	145
Output	LogSoftmax	(−1, 1, 256, 256)	0

Table A2 lists the network architecture and specific parameters of the multi-scale feature fusion module.

**Table A2:** Network architecture and specific parameters of the multi-scale feature fusion module.

Stage	Submodule/Layer (Type)	Input/Output Shape
Input	Encoder feature maps	(64, 128, 128), (128, 64, 64), (256, 32, 32)
Channel Alignment	3 × Conv2d (1 × 1, bias = False)	(64, 128, 128) → (128, 128, 128); (128, 64, 64) → (128, 64, 64); (256, 32, 32) → (128, 32, 32)
Channel Attention (per scale)	Global AvgPool → 2 × FC → Sigmoid	(128, H, W) → (128, H, W)
Spatial Attention (per scale)	AvgPool + MaxPool → Conv2d (7 × 7) → Sigmoid	(128, H, W) → (128, H, W)
Resolution Reconstruction	Bilinear Upsampling	(128, 128, 128); (128, 64, 64); (128, 32, 32) → (128, 256, 256)
Feature Concatenation	Channel Concatenation	3 × (128, 256, 256) → (384, 256, 256)
Fusion & Refinement—Stage 1	Conv2d (1 × 1, bias = False) + BN + ReLU	(384, 256, 256) → (128, 256, 256)
Fusion & Refinement—Stage 2	Conv2d (3 × 3, padding = 1, bias = False) + BN + ReLU	(128, 256, 256) → (64, 256, 256)
Fusion & Refinement—Stage 3	Conv2d (3 × 3, padding = 1, bias = False) + BN + ReLU	(64, 256, 256) → (16, 256, 256)
Output	Fused Feature Map	(16, 256, 256)

Table A3 lists the network architecture and specific parameters of PPO.

**Table A3:** Network architecture and specific parameters of PPO.

Module	Submodule/Operation	Input/Output Shape
Input State	Concatenation of multi-scale fused features, current change-probability map, confidence/uncertainty map, and spatial-consistency map	(19, 256, 256)
Shared Encoder (RLEncoder)	Conv2d (18 → 64, 4 × 4, stride = 2, padding = 1) + ReLU	(19, 256, 256) → (64, 128, 128)
	Conv2d (64 → 128, 4 × 4, stride = 2, padding = 1) + ReLU	(64, 128, 128) → (128, 64, 64)
	Conv2d (128 → 256, 4 × 4, stride = 2, padding = 1) + ReLU	(128, 64, 64) → (256, 32, 32)
	Conv2d (256 → 1, 1 × 1) + Sigmoid (Attention weighting)	(256, 32, 32) → (256, 32, 32)
Encoded Feature	Attention-weighted feature representation	(256, 32, 32)
Actor Head	ConvTranspose2d (256 → 128, 4 × 4, stride = 2, padding = 1) + ReLU	(256, 32, 32) → (128, 64, 64)
	ConvTranspose2d (128 → 64, 4 × 4, stride = 2, padding = 1) + ReLU	(128, 64, 64) → (64, 128, 128)
	ConvTranspose2d (64 → 32, 4 × 4, stride = 2, padding = 1) + ReLU	(64, 128, 128) → (32, 256, 256)
	Conv2d (32 → 6, 3 × 3, padding = 1)	(32, 256, 256) → (6, 256, 256)
Action Output	Pixel-wise discrete action index map; mapped to Δp ∈ {±0.1,±0.2,±0.5}	(6, 256, 256)
Critic Head	Conv2d (256 → 512, 3 × 3, padding = 1) + MaxPool2d(2 × 2) + ReLU	(256, 32, 32) → (512, 32, 32)
	AdaptiveAvgPool2d(1 × 1)	(512, 32, 32) → (512, 1, 1)
	Flatten → Linear(512 × 16 × 16→256) + ReLU → Linear(256 → 1)	(512, 1, 1) → (1,)
Value Output	State value estimation (V(s))	(1,)
