# Supplementary material for: Optimized Reinforcement Learning-Driven Model for Remote Sensing Change Detection
Source: J Imaging. 2026 Mar 19;12(3):139. doi: 10.3390/jimaging12030139 (PMC13027485; doi:10.3390/jimaging12030139)
Supplement: Supplementary file 1 [file jimaging-12-00139-s001.zip › jimaging-4118236-supplementary.pdf]

Section S1. Pilot Study on the Impact of NIR (PVCD)

S1.1. Objective

This pilot study aims to (i) estimate the potential quantitative benefit of adding a near-infrared (NIR) band on PVCD, and (ii) verify whether introducing NIR qualitatively changes the main RL refinement behavior (boundary refinement and pseudo-change suppression).

S1.2. Data Subset and Split

We constructed a small subset from PVCD consisting of **500 image pairs for training** and **100 image pairs for evaluation**. To prevent information leakage due to spatial autocorrelation, the evaluation pairs were selected from **independent sample areas** that were not used for training.

S1.3. Input Settings

Two input configurations were tested:

- **RGB-only:** three visible bands.
- **RGB+NIR:** RGB plus NIR band (720 nm, 750 nm, and 840 nm).
  All inputs follow the same preprocessing and normalization pipeline described in the main manuscript.

S1.4. Training Protocol

Backbone training: SiamU-Net was trained under both RGB and RGB+NIR settings using the same training protocol until convergence.

RL refinement training: The same RL module and PPO settings as in the main paper were used. For this pilot, the RL component was trained for **10,000 iterations** for both RGB-only and RGB+NIR settings, with the same discrete action set $A = \{\pm 0.1, \pm 0.2, \pm 0.5\}$.

S1.5. Quantitative Results (Table S1)

Table S1 summarizes the quantitative results on the held-out evaluation subset. Adding NIR yields a modest improvement for the backbone model. After incorporating the RL refinement module, the RGB-only setting achieves slightly higher final metrics than RGB+NIR in this limited pilot, while both settings maintain consistent RL refinement gains.

Table S1. Pilot comparison of RGB-only vs. RGB+NIR on a PVCD subset.

Input	Model	mIoU	F1
**RGB**	SiamU-Net	**70.25**	**79.64**
**RGB+NIR**	SiamU-Net	70.86	80.10
**RGB**	SiamU-Net + RL	72.15	81.24
**RGB+NIR**	SiamU-Net + RL	71.40	80.30

S1.6. Qualitative Results and Discussion (Figure S1)

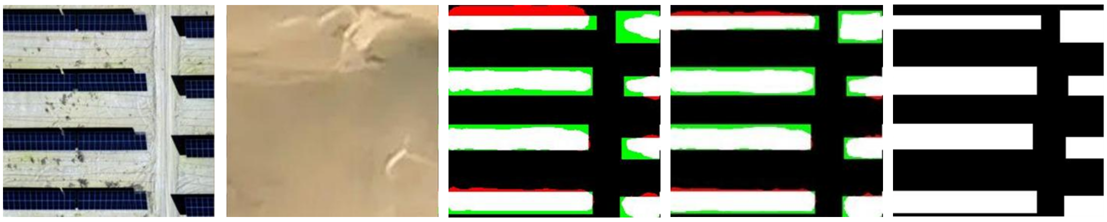

(a) Pre-event (b) Post-event (c) RGB+NIR (d) RGB+NIR+RL (e) GT

Figure S1. Qualitative comparison of RL refinement under RGB-only and RGB+NIR inputs on the PVCD pilot subset.

Figure S1 presents representative examples under RGB-only and RGB+NIR inputs. In both settings,

the reinforcement learning (RL) module consistently improves boundary delineation of change regions and suppresses pseudo-change responses, indicating that adding an NIR band does not fundamentally alter the core refinement behavior of RL. It is worth noting that, in this limited pilot, the RGB+NIR configuration yields slightly weaker quantitative results than RGB-only. We attribute this primarily to the increased complexity of the state representation after introducing an additional spectral channel, which typically requires more exploration iterations and/or dedicated tuning for the RL refinement stage. Since the purpose of this pilot is to assess whether NIR changes the qualitative RL behavior rather than to fully optimize the RGB+NIR setting, we used a fixed training budget of 10,000 iterations; under this limited budget, the RL exploration and convergence may be insufficient, leading to a small performance drop for RGB+NIR after applying RL.

Section S2. High-Resolution Mosaics and Zoom-in Boundary Verification

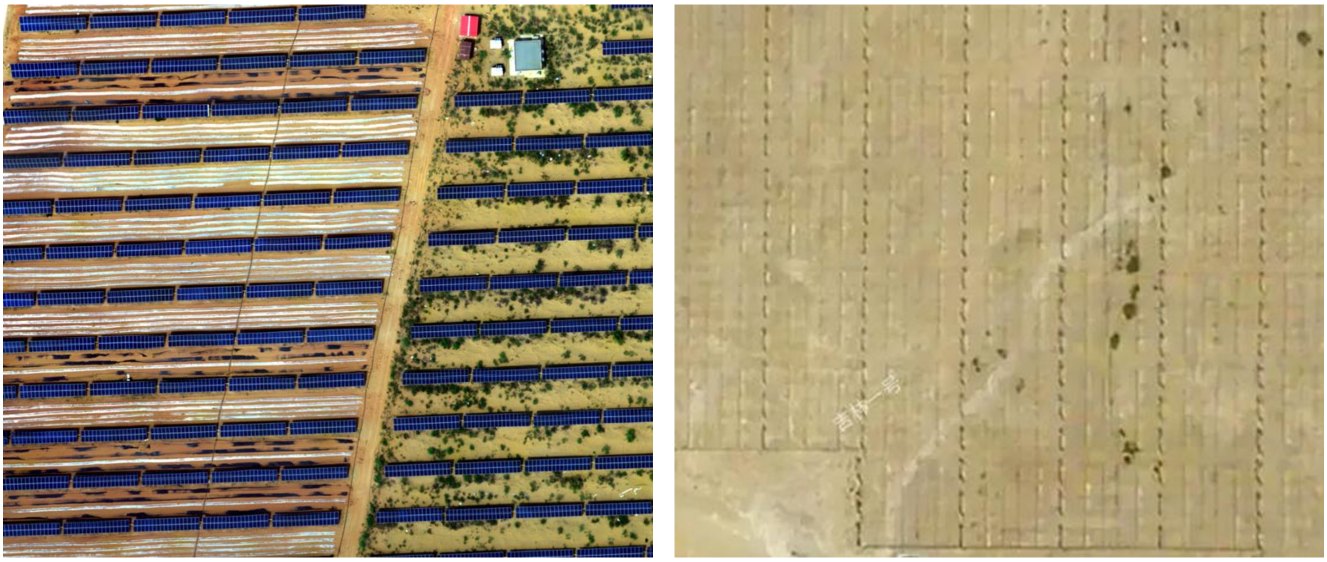

(a) Post -event (b) Pre-event

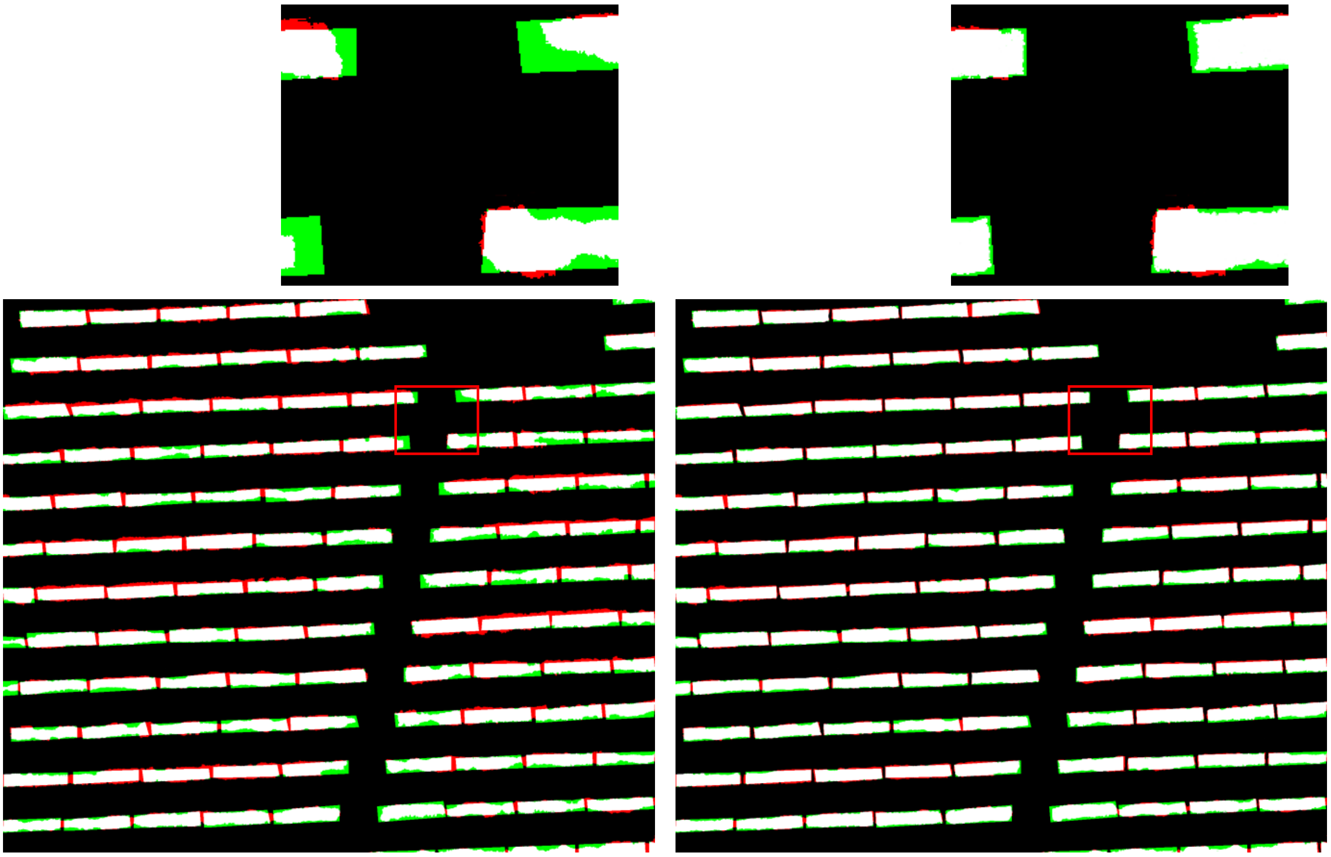

(c) Siam-UNet (d) Siam-UNet+RL

Figure S2. High-Resolution Mosaics and Zoom-in Boundary Verification.

Section S3. RL Gains Stratified by Dataset Type and Resolution Proxy

Table S2. RL gains stratified by dataset type and resolution/GSD proxy.

Dataset	Imaging condition proxy	Resolution/GSD (proxy)	Baseline mIoU	+RL mIoU	ΔmIoU	Baseline F1	+RL F1	ΔF1
**CDD**	Mixed-source general CD	~0.03–1.0 m	88.45	91.52	+3.07	93.64	95.29	+1.65
**SYSU-CD**	General CD (urban)	~0.5 m	72.68	75.22	+2.54	83.67	85.26	+1.59
**PVCD**	UAV PV-farm CD	1.0m	66.71	72.84	+6.13	79.49	84.10	+4.61
**BRIGHT**	Disaster-oriented CD (optical used)	~0.3–1.0 m	62.45	65.55	+3.10	70.05	72.10	+2.05
